# Measles outbreak investigation in Guji zone of Oromia Region, Ethiopia

**DOI:** 10.11604/pamj.supp.2017.27.2.10705

**Published:** 2017-06-09

**Authors:** Ketema Belda, Ayesheshem Ademe Tegegne, Amare Mengistu Mersha, Mekonnen Getahun Bayenessagne, Ibrahim Hussein, Belay Bezabeh

**Affiliations:** 1 World Health Organization, Oromia Region Technical Support Team; 2 World Health Organization Country Office, Addis Abba, Ethiopia; 3 Ethiopian Public Health Institute, Addis Ababa Ethiopia

**Keywords:** Measles, outbreak response, outbreak investigation, outbreak management, Ethiopia

## Abstract

**Introduction:**

Despite the increase of immunization coverage (administrative) of measles in the country, there are widespread outbreaks of measles. In this respect, we investigated one of the outbreaks that occurred in hard to reach kebeles of Guji Zone, Oromia region, to identify the contributing factors that lead to the protracted outbreak of measles.

**Methods:**

We used a cross-sectional study design to investigate a measles outbreak in Guji zone, Oromia region. Data entry and analysis was performed using EPI-Info version 7.1.0.6 and MS-Microsoft Excel.

**Results:**

In three months' time a total of 1059 suspected cases and two deaths were reported from 9 woredas affected by a measles outbreak in Guji zone. The cumulative attack rate of 81/100,000 population and case fatality ratio of 0.2% was recorded. Of these, 821 (77.5%) cases were < 15 years of age, and 742 (70%) were zero doses of measles vaccine. Although, all age groups were affected under five years old were more affected 495 (48%) than any other age groups. In response to the outbreak, an outbreak response immunization was organized at the 11th week of the epidemic, when the epidemic curve started to decline. 6 months to14 years old were targeted for outbreak response immunization and the overall coverage was 97 % (range: 90-103%). Case management with vitamin A supplementation, active case search, and health education was some of the activities carried out to curb the outbreak.

**Conclusion:**

We conclude that low routine immunization coverage in conjunction with low access to routine immunization in hard to reach areas, low community awareness in utilization of immunization service, inadequate cold chain management and delivery of a potent vaccine in hard to reach woredas/kebeles were likely contributed to the outbreak that's triggered a broad spread epidemic affecting mostly children without any vaccination. We also figured that the case-based surveillance lacks sensitivity and timely confirmation of the outbreak, which as a result outbreak response immunization were delayed. We recommend establishing reaching every child (REC) strategy in Guji zone with particular emphasis too hard reach areas to enhance the current immunization service, and furthermore to conduct data quality self-assessment or cluster coverage survey to verify the reported high vaccination coverage in some kebeles. We also recommend conducting the second opportunity as a form of supplemental immunization activities in 2-3 year interval or consider the national second dose introduction in the routine immunization system to improve population immunity. We further recommend that there is a need to boost the sensitivity of case-based surveillance system to be able to early detect, confirm and react to future epidemics.

## Introduction

Despite the availability of safe and effective measles vaccine since 1963, measles still remains a public health concern. However, with the introduction of the measles accelerated control strategy and mortality reduction goal, the mortality has decreased in both developed and developing countries. In 2001, countries in the World Health Organization (WHO) African Region started implementing the regional measles mortality reduction goal to reduce the estimated number of measles deaths by half in 2005 compared to 1999 [1]. The measles mortality reduction strategies include the following three elements: 1) improving routine measles vaccination coverage; 2) providing a second opportunity for measles vaccination through supplemental immunization activities (SIAs); and 3) monitoring the impact of vaccination activities through case-based measles surveillance, and improving measles case management [1]. To this end, the assessment conducted in 2010 indicated that global mortality from measles has been reduced by 74% of the global estimate in 2000, and 85% reduction in the African region has been documented [2]. In 2002 Ethiopia adopted the African regional accelerated measles morbidity and mortality reduction goal to reduce measles mortality, including the pre-elimination goal which ended in 2012 [3]. Since the routine immunization started in 1980 measles vaccination is provided at 9 months of age for infants throughout the country. On top of this, measles catches up campaigns were conducted for all <15 years old in phase manner at the beginning of the accelerated control program, and in 2-3 years interval supplemental immunization activities was conducted. However, despite all these efforts, the estimated measles first dose (MCV1) coverage in Ethiopia was 56% in 2010 and 57% in 2011 and the percentage of woredas, which is the lowest administrative units reporting ≥80% MCV1 coverage was 45% in 2010 and 43% in 2011 [4]. As a result of the low population immunity and buildup of the susceptible population, widespread measles outbreaks were occurring in 2015 throughout the country, and the numbers of districts affected by measles epidemic were more than 280 as per the weekly measles report. The objective of this investigation is to assess the magnitude and identify the contributing factors for the measles outbreak in Guji and generate evidence for the prevention and control of future outbreaks.

## Methods

**Study Area:** this investigation was conducted in Guji zone in the Oromia region, which is located in the southeastern part of Ethiopia. Oromia region is the most populous region in the country, with a population of 39 million as per the 2007 census, and a growth rate of 3.8% per annum. The Region has 20 zones characterized by varying ecologies, climates, and populations. The zones are divided into 246 woredas and, which is further divided into kebeles. Guji zone is administratively the zone is divided into 14 rural and two town administrations. The total population of the zone is estimated to be 1,787,760 according to the 2007 census and housing projection.

**Study design:** we conducted a cross-sectional study, including a review of the medical records and interviews.

Study subjects: all children affected by measles were subject of the study. A line list and a semi-structured questionnaire were used to obtain information on the risk factors associated with the outbreak. Demographic information was collected on patient characteristics, including date of onset, date of visit to health facilities, outcome, and vaccination status. Additional data were collected on cold chain management and patient management. We also conducted house-to-house and community active case searches for unreported cases of measles.

**Laboratory methods:** serum specimens were collected from five suspected cases and sent to the national measles laboratory for IgM test and a test done as per the global and national guidelines.

**Data analysis:** data were entered and analyzed using Epi-Info7 version 7.1.0.6 and Microsoft Excel. All data were cleaned for completeness before analysis. Attack rates, vaccination status, and case fatality rates were calculated and results were presented using graphs and tables.

### Case definitions

**Suspected measles outbreaks:** defined as the occurrence of five suspected measles cases in one month in a defined geographic area such as a kebele, woreda or health facility catchment.

**Confirmed measles outbreaks:** defined presence of three or more laboratory-confirmed measles cases in a one-month time per kebele or woreda or health facility.

**A suspected measles case:** any person with generalized maculopapular rash and fever plus one of the following: a cough or coryza (a runny nose) or conjunctivitis (red eyes).

**A laboratory confirmed cases:** a suspected case, which has laboratory results, indicating infection (IgM positive or isolated for a measles virus).

**Not eligible:** children age < 9 months affected by measles outbreak or not eligible for vaccination.

## Results

**Figure 1:**
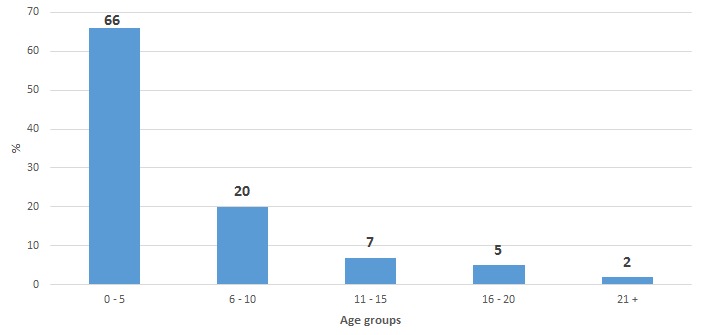
Distribution of Measles Cases by age group, Guji zone, Ethiopia, June 2015 (n=1059)

From January 8 to March 9, 2015, a total of 1059 suspected measles cases and 2 deaths (Case Fatality Rate=0.2%) were reported from nine Woredas in Guji zone. Of those, 5 (0.47%) were confirmed by laboratory investigation (IgM positive), while the rest were epidemiologically linked and clinically compatible cases. The median age was 36 months ranging from 3 to 360 months (interquartile range 24 to 72 months) and there were 515 (51%) females and 544 (49%) males (Table 1). Among the 1,059 cases, 698 (65.9%) were <5 years old (Table 1 and Figure 1). The age-specific attack rates vary and were 17/100,000 and 36/100,000 populations for the age group <1 year and 1-4 years respectively, while the overall attack rate was 81/100,000 population. In total nine woredas (9/16) and 118 kebeles (118/ 369) were affected, and the attack rate varies from woreda to woreda and ranged from 222 cases per 100,000 populations in Dama district to 11 cases per 100,000 in the Hamble Wamena woreda (Table 2).

**Table 1:** Vaccination status of children affected by measles in Guji zone, Ethiopia (n=1059)

Age		Sex		Vaccination status			specimen collected		Result (+ve)
	Male	Female	Total	Not vaccinated	Not eligible	Vaccinated (1+dose)	Yes	No	
0-5	329	369	698	495	79	123	1		1
6-10	42	33	216	184	0	29	2		2
11-15	34	14	75	63	0	5			
16-20	14	8	48	34	0	5			
21+	96	120	22	16	0	1	1		1
**Total**	**515**	**544**	**1059**	**792**	**79**	**163**	**5**		**5**

**Table 2:** Administrative immunization coverage and measles attack rate, Guji zone

Name of woreda	2009	2010	2011	2012	2013	2014	Average Immunization coverage	Attack rate/100,000 population in affected woredas
Adola Rede	66	66	78	71	56	90	71.2	119
Adola Wayu	88.6	86.9	90	53	56	109	80.6	NA*
Anasora	65	71.1	113	74	95	134	92.0	124
Bore	63	60.8	63	79	71	129	77.6	61.9
Dama	89.1	66.6	104	91	89	115	92.5	222
Girja	65.5	97.8	123	61	99	86	88.7	19.3
Goro Dola	92.5	92.6	99	85	89	115	95.5	NA*
H/ Wamana	134.7	76.8	82	107	110	121	75	10.9
Kercha	87.1	73.8	63	104	79	110	86.2	49
Liban	98	75	85	84	87	93	87.0	NA*
Negele T	101.6	116.3	138	153	175	237	153.5	NA*
Odo Shakiso	112	71.9	105	63	88	121	93.5	106
Sababoru	123.6	117.2	100	81	100	108	105.0	NA*
Shakiso T	NA	NA	NA	NA	79	76	25.8	NA*
Uraga	93.1	81.9	99	97	91	95	92.8	85
Wedera	75.8	74	123	72	58	93	82.6	NA*
Zone	91	78.9	104	87	87	112	93.3	81.2

* None affected woredas

**Figure 2:**
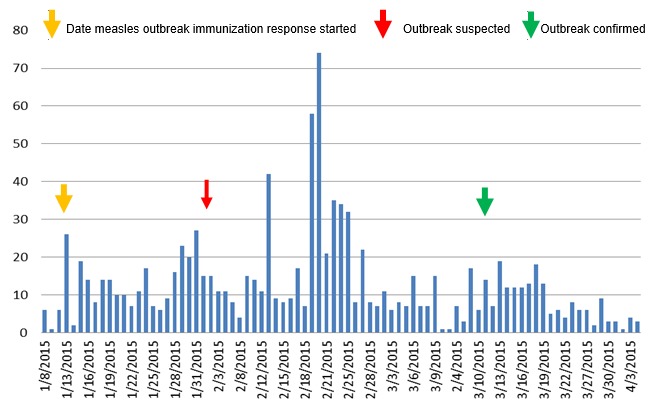
Epi-curve of Measles cases by date of onset, Guji zone, Ethiopia, June 2015 (n=1059)

**Figure 3:**
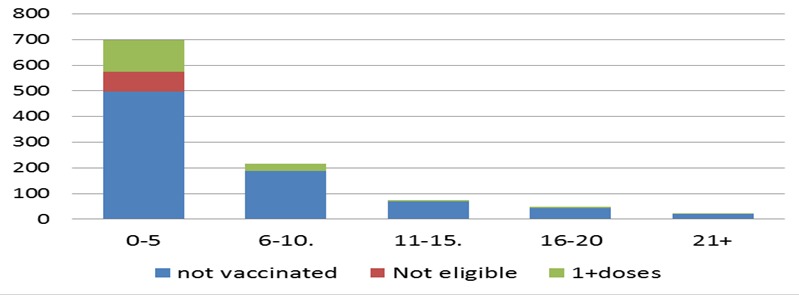
Vaccination status of measles cases by age group Guji, 2015 Oromia Region (n=1059)

The index case date onset was on January 8, 2015, and was reported from Braga district, Haru kebele of Guji zone, and seen at health facility level on January 12, 2015. The cases started to build up slowly with a fluctuating trend and reached a peak on February 20, 2015, which then started to decline shortly although it showed an inconsistent trend (Figure 2). Regardless of its remoteness and difficulties in providing immunization service, the review of immunization coverage for the last 6 years indicate an overall coverage of 93.5%, while it varied from woreda to woreda (ranged 70-112%). On the other hand of those affected children, 792 (75%) of them were with zero doses, while 163 (16%) of the cases had received only 1-2 doses of measles, and interestingly of those cases unvaccinated, 496(71%) were < 5 years old (Table 2, Figure 3).

**Figure 4:**
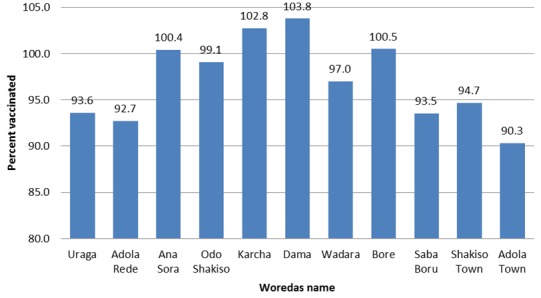
Outbreak immunization response coverage by Woreda, Guji zone, Ethiopia

The outbreak occurred in remote kebeles with many hours walk on foot from the main road which makes it difficult to provide routine immunization service. Despite, the difficulties an outbreak response immunization was conducted starting on March 15, 2015, targeting children from 6 months to14 years, Although, the disparity between woredas exist 97% administrative coverage was achieved, while it ranges from 90.3% to 103% (Figure 4).

The nomadic nature of the population and hard to reach areas challenged the routine immunization delivery system and during an outbreak investigation, it was witnessed that health posts in outbreak affected areas suffered from a lack of constant delivery of potent vaccine and regular maintenance of cold chain equipment.

## Discussion

Our outbreak investigation showed that more male children were affected than females, and also more <15 years of age were primarily affected by the outbreak. A high proportion of unvaccinated children (77%) were affected in this outbreak indicating the low level of population immunity despite high coverage reported in the zone. The zone like the rest of the country had conducted an initial catch-up campaign for those who are <15 years old and periodic SIAs for children <5 years old with an interval of 2-3 years. The outbreak affected all age groups, even older than 15 years, which may indicate the persistent low routine immunization coverage over several years and the accumulation of the susceptible population in the older age group that may have led to the current outbreak. Inadequate and poor cold chain management system coupled with hard to reach kebeles makes difficult to provide potent vaccine in routine immunization service likely also contributed to the outbreak. While the zone made some prominent progress in improving routine immunization, there is still pocket and hard to reach areas that are difficult to reach and provide routine immunization despite the introduction of routine immunization for over three decades. The number of unvaccinated children in our findings was higher than (75%) the studies reported from Marshall Islands (59%), and the study conducted in Rio de Janeiro, which was 7.2% in < 1 years old [5–7].

We found also a higher attack rate than the attack rate of measles outbreak recorded nationally, 4.1 per 100,000 population, in 2008 [8]. We also found the disparity in the attack rate among woredas. This may be due to delayed detection and confirmation of the epidemic, which leads to a delayed response to the epidemic. On the other hand, the higher attack rate may be due to the build-up of the susceptible population which may have contributed the spread of the disease faster than esxpected. The disparity of attack rate may be the reflection of routine immunization as the performance of woredas in regard to routine immunization is different between woredas. The response vaccination conducted at the time of the decline of Epi-curve may have had little or no impact on the overall epidemic control since many of the children may have been already exposed or contracted the infection and may have recovered from measles illness. The highest attack rate (219 cases per 100,000) was observed among children <1 year old, which is the reflection of weak routine immunization in the affected woredas, and the finding is similar to a study condcuted in S.Africa [9]. The attack rate in our finding is higher than the cumulative attack rate, reported(37/100000) in South Africa's and other studies conducted including Shimada district (41/100000) in Ethiopia [9, 10]

Additionally, a relatively large proportion of cases <9 months were affected by the epidemic, which creates a concern as regard to the age at which vaccination should start. This may be due to contributing factors like malnutrition, and it may also be that children of this age group may have no maternal antibody at the very begging which may indicate a long standing problem with measles vaccination. The case fatality rate in this study was low (0.2%), while in general measles case fatality can reach from 3 - 5% in developing countries and may goes up to 10% in closed outbreaks [11] when it is compounded by malnutrition [12]. The lowest case fatality rate in this study may be due to the fact that death at the community level is not registered and this only includes health facility deaths. The study conducted in Simada district in Ethiopia indicated a higher CFR rate (13.4%), including the study done on hospital admitted cases in Zimbabwe, Kenya, and Niger [13–15]. However, our findings are similar with that of Sudan (0.9%) [16].

To prevent measles outbreaks or interrupt transmission and to enhance elimination of measles, 95% population immunity is needed. However, the administrative coverage of measles vaccination in the zone under review range from 78.9-112% for the last six years with great disparity between woredas, which indicates suboptimal population immunity to prevent an outbreak. The absence of functional fridge at health post level and hardship topography setup coupled with long travel distance to get the vaccine from the health center may have contributed to the potency of the vaccine, and as a result, it could be the contributing factors for low population immunity while high coverage reported in some woredas where the epidemic occurred.

The limitation of our study may be incompleteness of data and the inclusion of non-measles cases that doesn't fulfill the case definitions as line list were collected by health workers at a lower level. Additionally, death recorded only captures those deaths that occurred at health facility level, and community death is not reported.

## Conclusion

We conclude that, almost all cases are reported by health centers and hospitals in SNNPR, Amhara, Tigray and Oromia, the bigger regions of the country. Private health facilities in Addis Ababa and health posts in Benishangul-Gumuz, Somali and Afar have made significant contributions in AFP case notification and reporting. Cases reported by health posts are relatively lower quality. We recommend that all potentially reporting sites should be exhaustively identified, prioritized and feasible strategies designed to be regularly supported for quality case detection, investigation and reporting. Private health facilities in major towns of each region should also be targeted for surveillance strengthening. Furthermore, a review of the case investigation tools to determine the contribution of traditional sites in case reporting would increase the evidence for further strengthening of the surveillance system at community level.

### What is known about this topic

- Measles accounts for much of the vaccine-preventable disease burden in Ethiopia;
- Despite the increase in measles coverage, frequent outbreaks of measles continue to occur in Ethiopia;
- Measles outbreaks responded with immunization and surveillance activities to manage outbreaks at the local level in Ethiopia. Timely responses to outbreaks usually not meet in Ethiopia.

### What this study adds

- The study provides valuable information on the measles outbreak in very hard to reach and pastoralist zone;
- The study provides also information on the impact of early immunization response to control outbreaks of measles;
- The study also indicates the need to have sensitive and strong surveillance to timely detect and respond to an outbreak of measles.

## Competing interests

All authors declare no competing interests. The views expressed in the perspective articles are those of the authors alone and do not necessarily represent the views, decisions or policies of the institutions with which they are affiliated and the position of World Health Organization.
